# De‐mystifying the “Mixifusor”

**DOI:** 10.1111/pan.14039

**Published:** 2020-11-04

**Authors:** Anthony R. Absalom, Ann E. Rigby‐Jones, Andrew R. Rushton, J. Robert Sneyd

**Affiliations:** ^1^ University Medical Center Groningen University of Groningen Groningen The Netherlands; ^2^ Faculty of Health: Medicine Dentistry and Human Sciences University of Plymouth Plymouth UK; ^3^ Department of Anaesthesia University Hospitals Plymouth Plymouth UK

**Keywords:** clinical pharmacology, pharmacokinetics, propofol, remifentanil, safety, target‐controlled infusions

## Abstract

Total intravenous anesthesia (TIVA) using a mixture of propofol and remifentanil in the same syringe has become an accepted technique in Pediatric Anesthesia. A survey by a group of respected UK anesthetists demonstrated a low incidence of serious complications, related to the pharmacology and dose of the drugs. However, a current guideline for the safe use of TIVA recommends against this practice. Pharmaceutical concerns include the physical stability of the emulsion when remifentanil is mixed with propofol; changes in drug concentration over time; nonuniform mixing of propofol and remifentanil; the risk of bacterial contamination; and the potential for drug administration errors. Propofol and remifentanil have markedly different pharmacokinetic profiles. When remifentanil is mixed with propofol and delivered as a target‐controlled infusion (TCI) of propofol, remifentanil delivery is not target‐controlled but passively follows the variable infusion rates calculated by the syringe driver to deliver predicted plasma or effect‐site concentrations of propofol. The pharmacokinetic consequences can be illustrated using pharmacokinetic modeling similar to that used in TCI pumps. The clinical consequences reflect the dose‐dependent pharmacodynamics of remifentanil. Increasing the target propofol concentration produces a rapid increase and peak in remifentanil concentration that risks apnea, bradycardia, and hypotension, especially with higher concentrations of remifentanil. The faster decline in remifentanil concentration with falling propofol concentrations risks inadequate narcosis and unwanted responses to surgical stimuli. Remifentanil delivery is inflexible and dosing cannot be adjusted to the clinical need and responses of individual patients. The medicolegal considerations are stark. In UK and EU Law, mixing propofol and remifentanil creates a new, unlicensed drug and the person mixing takes on the responsibilities of manufacturer. If a patient receiving anesthesia in the form of a mixed propofol‐remifentanil infusion suffered a critical incident or actual harm, the clinician's practice may come under scrutiny and criticism, potentially involving a legal challenge and the Medical Regulator.

## 1 INTRODUCTION

Older anesthetists may remember the crunch of precipitating thiopental caused by injecting succinylcholine into an un‐flushed cannula. Trial and error (lots of errors…) showed that precipitation may follow in any number of unlicensed drug mixtures with succeeding generations repeating the mistakes of their predecessors.^1^, ^2^ Naïvely, anesthetists may assume that the absence of crystals or of any other changes visible to the naked eye implies compatibility but this is not necessarily the case. Ever since it first became available for use in clinical practice, anesthetists have experimented with the addition of diluents and other drugs to the propofol formulation. As propofol is presented as a sterile oil in water emulsion, the addition of diluents and other drugs poses several problems. The opacity of the propofol emulsion likely conceals any evidence of crystallized drug, and important changes in the lipid emulsion characteristics are not visible to the naked eye.

The most common additive is lidocaine, which reduces pain on injection.^3^, ^4^ Anesthetists have also added other drugs such as alfentanil or ketamine to propofol, because they wish to infuse propofol and the added drug, using a single infusion pump.^5^, ^6^, ^7^ Reasons for using a single pump to infuse two or more drugs range from convenience to a lack of resources.

Total intravenous anesthesia (TIVA) is growing in popularity among pediatric anesthetists, often using a mixture of propofol and remifentanil.^8^ There are several reasons why pediatric anesthetists might be tempted to use this technique. A second infusion pump is not always readily available, even in high resource countries, and administration of remifentanil from a separate syringe and line increases cost. Pediatric surgery operating schedules commonly list several short duration procedures in quick succession, and mixing of two drugs in one syringe saves (a small amount of) time. Finally, in small children, it is desirable to limit administered fluid volumes, and mixing remifentanil in the propofol emulsion reduces the volume of carrier fluid.

A recent guideline for safe use of total intravenous anesthesia (TIVA) noted the increasing prevalence of mixing of drugs among pediatric anesthetists, but recommended against this practice, particularly when propofol is administered by target‐controlled infusion (TCI).^9^ The reasons for caution quoted in the guideline are largely consistent with those mentioned in a previous editorial on mixing,^10^ which center mostly on medio‐legal and safety concerns. In an attempt to demonstrate the safety of mixing propofol and remifentanil, a group of respected UK pediatric anesthetists performed a service evaluation of the safety and efficacy of this technique in almost 900 patients, and presented their findings in this journal.^11^

Bagshaw and colleagues prospectively collected data from 880 patients, the majority of whom were healthy (88% ASA 1‐2) and younger than 11 years of age (76%), undergoing a wide range of procedures (16 specialties in total). In the majority of cases (79%), the remifentanil concentration was 5 mcg/mL in the 1% propofol emulsion (or its equivalent mixture, remifentanil 10 mcg/mL in 2% propofol), but there were also instances when 2.5, 10 and 20 mcg/mL (or equivalent) were used. In brief, there were 224 complications during 159 anesthetics. The majority were minor and expected, such as movement or coughing, whereas the incidence of events that were serious, related to the pharmacology of the drugs but unexpected and requiring intervention, was only 1.7%. With a sample size of 873 patients, the 95% confidence interval for this proportion is approximately 1%‐2.8%. Complications were more likely to occur when the remifentanil concentration in the syringe was ≥ 10 mcg/mL.

Does this result show that it is acceptable for you to try out this technique in your hospital? A clinical practice is traditionally regarded as acceptable and defensible if a body of respected colleagues uses it, and this is certainly the case here. Do these data prove that the technique is safe? This is a tough question to answer. The confidence interval mentioned above certainly falls well below the published incidence of severe complications found in the APRICOT study (3.3% in the UK, 5.2% across all involved countries).^12^ On other hand, safety is usually only regarded as proven once a drug or technique has been studied in thousands of patients, and under similar conditions. The investigators are all highly experienced experts, and they present the results of an observational study in what is essentially a convenience sample. If you are considering trying this practice in your own hospital, then it would be worthwhile spending some time in the company of an expert to learn the technique.

Moreover, this study simply looked at the incidence of clinical adverse events. Issues that the study did not address, but which should be borne in mind include physicochemical stability and compatibility of the mixture, other safety aspects including bacterial contamination and drug error, differences in pharmacokinetics of the two drugs which can cause specific problems when infusion rates are high or low, and finally medicolegal matters. Some of these have been discussed previously,^10^ but we feel that their importance is sufficient to warrant a reprise.

## 2 PROPERTIES OF EMULSIONS AND WHAT MIXING WILL DO TO THEM

Ordinarily an oil in water emulsion is in a dynamic state. Droplets coalesce producing fewer but larger ones. Ultimately visible “creaming” may occur. Historically, this was visible in glass bottles of milk where over time fatty droplets coalesced and then floated to the top of the aqueous component, forming a layer of cream.

The original commercial propofol formulation, Diprivan® (AstraZeneca, Macclesfield UK), contains propofol emulsified in 10% Intralipid® (Kabi, Munich, Germany), which contains soya oil (100 mg/mL), egg yolk lecithin (12 mg/mL), and glycerol (22.5 mg/mL). This emulsion is subjected to homogenization producing droplets of 150‐300 nm diameter which are stabilized and emulsified by the egg yolk lecithin.^13^ In addition to providing a physical‐chemical barrier at the surface of each droplet, the 3 long‐chain acids of egg lecithin support a stable negative electrostatic charge, the zeta potential which causes droplets to repel each other thereby maintaining their dispersal and subsequently the stability of the emulsion.

Addition of an ionized drug such as lidocaine hydrochloride to a propofol emulsion provides a charge carrier allowing dispersal of the zeta potential, increasing droplet size and potentially precipitating creaming. Thus, the datasheet (summary of product characteristics, SPC) for propofol specifies that propofol emulsion diluted with 5% dextrose solution (uncharged) or supplemented by alfentanil (mostly unionized) may be considered stable over a 6‐hour period, whereas propofol with added lidocaine (mostly ionized) must be used immediately.^14^ Admixture of remifentanil is not mentioned.

## 3 (IN)STABILITY OF REMIFENTANIL AND PROPOFOL IN A MIXTURE

Stewart and colleagues studied the stability of propofol and remifentanil concentrations when the drugs are mixed, for 36 hours.^15^ Using Diprivan 1% they prepared mixtures containing 5 mcg/mL and 50 mcg/mL, in standard plastic syringes, and in PVC bags. For each mixture, control solutions were also prepared—that is, remifentanil only (5 mcg/mL and 50 mcg/mL solutions) and propofol only (10 mg/mL)—in plastic syringes and PVC bags. In the 5 mcg/mL admixtures in plastic syringes and PVC bags, remifentanil concentrations decreased significantly over time (by 8% and 12%, respectively, within the first hour), whereas in the 50 mcg/mL admixtures, the remifentanil concentrations remained stable. The control solutions all remained stable, except for propofol in PVC bags, where the propofol concentrations declined slowly over time, presumably because of interaction between the propofol and the PVC of the bag.

In the Bagshaw study,^11^ the majority of clinicians used admixtures with 5 mcg/mL of remifentanil, a concentration in which the remifentanil concentration declines over time, but which is associated with a lower incidence of adverse events. Given the fact that the median duration of administration was 32 minutes, the decline in remifentanil concentration over this time is unlikely to have been clinically significant.

## 4 LAYERING ISSUES/ MIGRATION OF REMIFENTANIL WITHIN MIXTURES

When two drugs are mixed and then infused, the infusion rates of each drug will only be and remain consistent with expectations if each drug mixes uniformly throughout the mixture. O’Connor found that this is not the case for propofol and remifentanil.^16^ They added remifentanil to propofol 1% in a vertically mounted syringe to produce mixtures containing remifentanil 25, 50, and 100 mcg/mL. Immediately after mixing, and for the duration of their experiment (300 minutes), the remifentanil concentrations were significantly higher in the uppermost portions of the mixture. For propofol, the gradient was in the opposite direction. This effect was most pronounced for the 25 mcg/mL mixture. Ten minutes after mixing the drugs, the concentrations at the top and bottom of the mixture were 16 and 4 mcg/mL for remifentanil, and 5.3 and 8.6 mg/mL for propofol, respectively. Their results showed that in addition to the large gradients, the concentrations of both drugs were significantly lower than expected.

In the Bagshaw study,^11^ anesthetists used a TCI pump to administer the mixture, and on all current TCI pumps the syringe is mounted horizontally. Although it is likely that the layering mentioned above is less of a problem with a horizontally mounted syringe, this has not been evaluated in a published study.

## 5 BACTERIAL CONTAMINATION

Propofol emulsions are sterile but strongly support bacterial growth at room temperature. In most countries, the emulsion does not contain an antiseptic (the United States in an exception). The triad of inadvertent contamination (during preparation), a period of incubation and subsequent administration, led to very serious and sometimes catastrophic clusters of infections in the United States.^17^, ^18^ Since 1996, propofol formulations sold in the United States have contained either EDTA or sodium metabisulphite (which apparently do not alter the safety or stability of the emulsion).^18^ European anesthetists rely on hand hygiene and aseptic technique.

When remifentanil is added to propofol, it must first be dissolved, as it is presented as a powder, and this step and the subsequent addition to the propofol present opportunities for contamination.

## 6 LACK OF STANDARDIZATION; RISK OF DRUG ERRORS

Propofol/opioid mixtures are visibly indistinguishable from the unadulterated injectate thereby generating opportunities for drug administration errors. Although strict regulations require the proper labeling of drugs prepared in operating theaters, these are not universally followed. Further, the additive increases the net volume of the mixture thereby decreasing the concentration of propofol in the resulting mixture. While the dilution caused to a 47.5‐mL syringe of 1% propofol injection by the addition of remifentanil 250 mcg in 2.5 mL (made by diluting remifentanil 1 mg to 10 mL) is probably insignificant—the resultant propofol concentration is 0.95% instead of 1.0%—this effect may become relevant if larger volumes of injectate are added to propofol solutions.

## 7 LEGAL CONSIDERATIONS

In the UK, the Medicines and Healthcare products Regulatory Agency clearly describes the legal position of mixing of drugs as follows:

Under current UK and European legislation, except in very restricted circumstances, mixing drugs together, where one is not a vehicle for the administration of the other, creates an unlicensed medicine. The person undertaking this preparation, unless an exemption applies, must hold a manufacturer’s licence.^19^

The above implies, in theory at least, that the mixer becomes the manufacturer (of the mixture) and is therefore legally liable for the consequences of its administration. This is a daunting prospect, albeit a theoretical one. Furthermore, as these mixtures constitute unlicensed medicines, and/or off‐label use of licensed medicines, this adds to the responsibilities of the clinician. Not only does the UK General Medical Council advise doctors against using drugs in a nonstandard and unapproved manner, it also specifically advises clinicians about the additional requirements for documentation and consent.^20^

## 8 PHARMACOKINETIC INCOMPATIBILITY

The pharmacokinetics of propofol and remifentanil can be described by multi‐compartment mammillary models.^21^ Although these models contain some assumptions which are demonstrably invalid (eg, that mixing within the central compartment is instantaneous after administration), George Box's remark that "all models are wrong, but some are useful" certainly applies.^22^ As these models are ubiquitous in anesthesia teaching and research and are widely deployed in TCI systems,^23^ we can turn to them to describe the likely consequences of co‐administering propofol and remifentanil, whose pharmacokinetics and pharmacodynamics differ. In the Bagshaw study,^11^ the responsible anesthetists most commonly administered a mixture containing 5 mcg/mL of remifentanil in propofol 1% using a TCI pump programmed with the Paedfusor^24^ pharmacokinetic model for propofol, with a median starting target concentration of 4 mcg/mL and a median end concentration of 2.9 mcg/mL.

**Figure 1:**
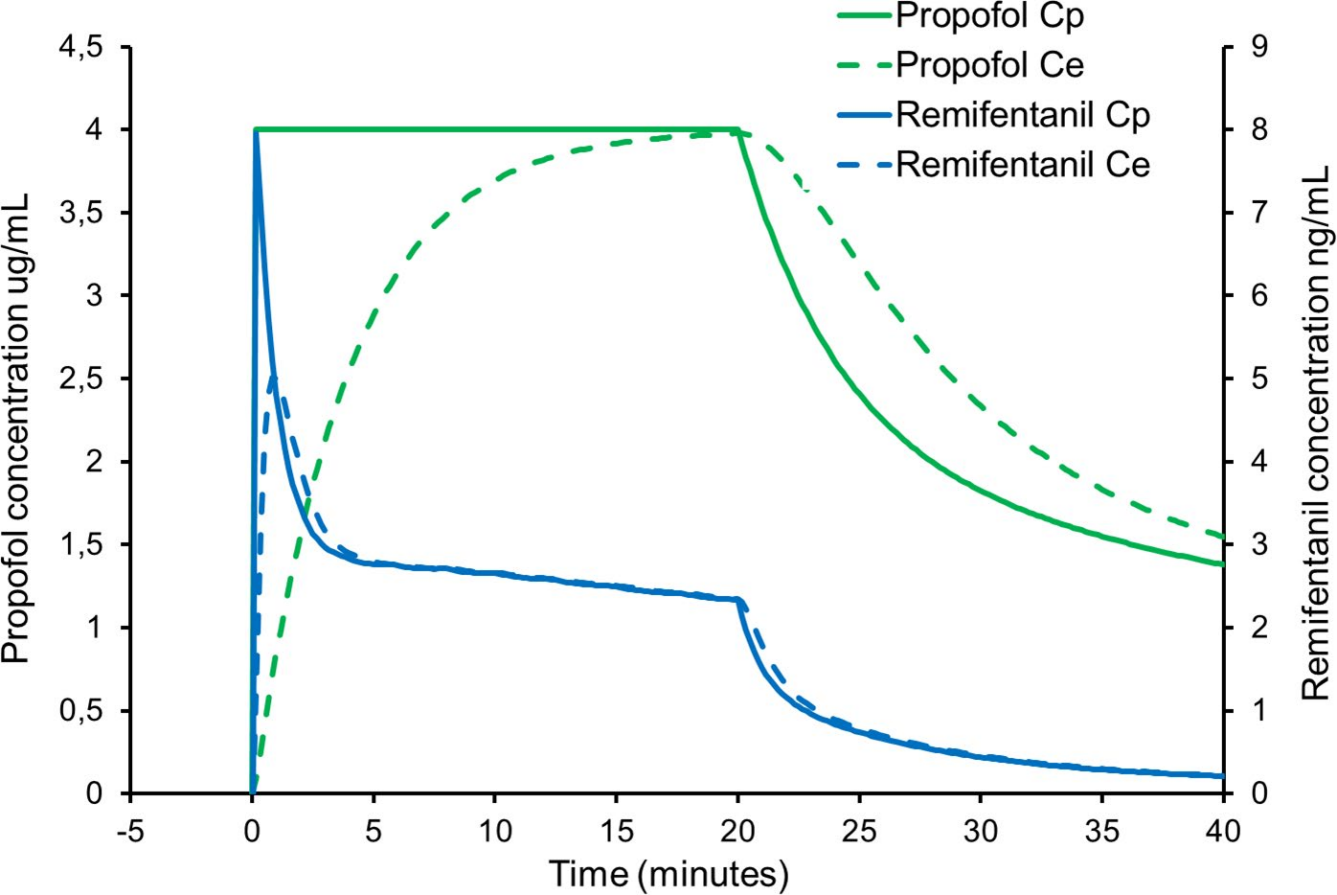
Simulated TCI of propofol (target plasma concentration 4 mcg/mL for 20 minutes) for a 10‐year‐old male, weight 32 kg and height 140 cm using Stanpump software. Remifentanil has been added to the propofol to yield a concentration of 5 mcg/mL. Predicted plasma propofol concentration (solid green line) and effect‐site concentration (dashed green line). Predicted plasma remifentanil concentration (solid blue line) and effect‐site concentration (dashed blue line). The Paedfusor and Eleveld pharmacokinetic sets were used for propofol and remifentanil, respectively. Predicted effect‐site remifentanil concentration overshoots when the infusion is started

**Figure 2:**
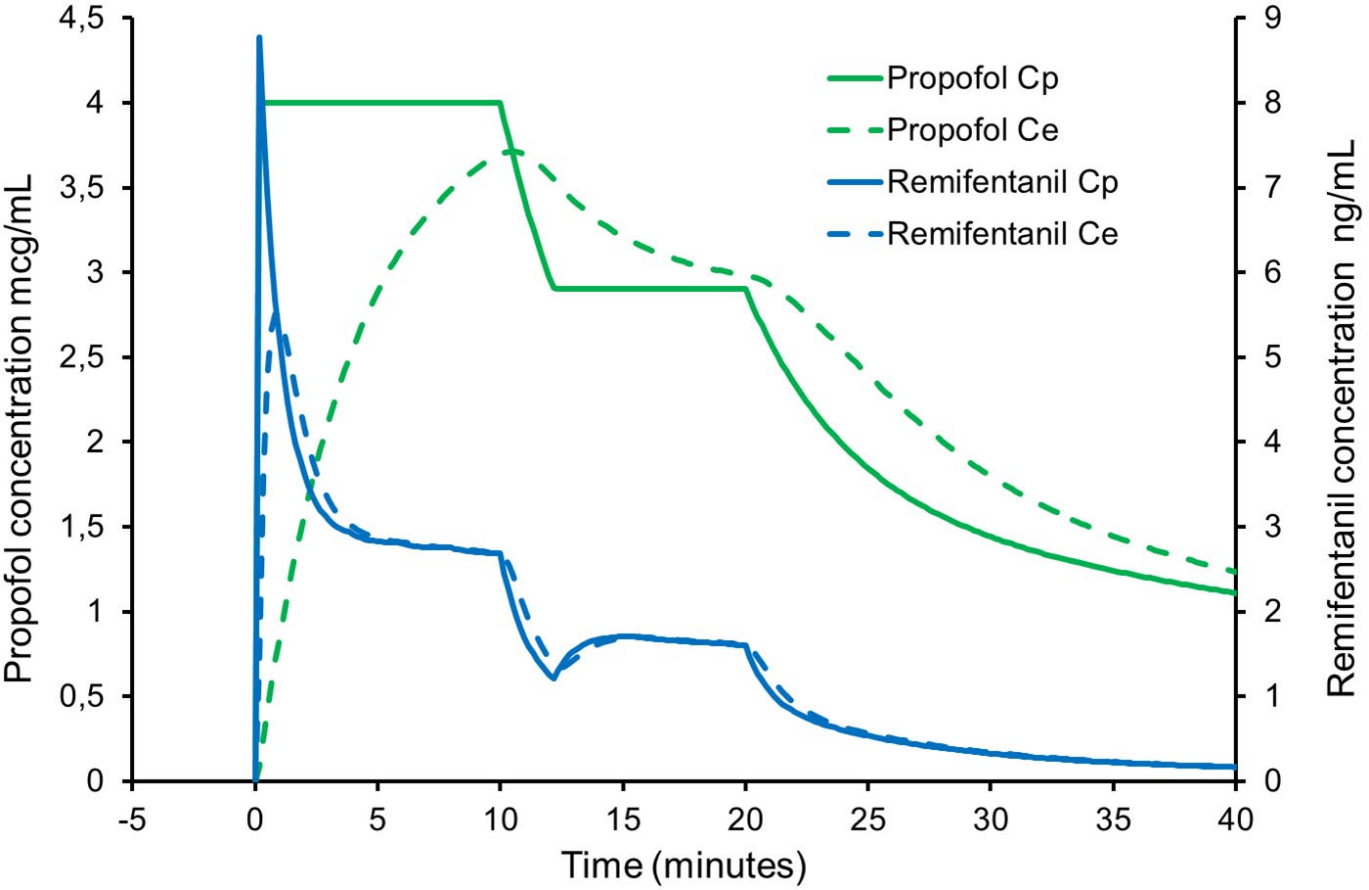
Simulated TCI of propofol (target plasma concentration 4 mcg/mL for 10 minutes and then 2.9 mcg/mL for 10 minutes) for a 10‐year‐old male, weight 32 kg and height 140 cm using Stanpump software. Remifentanil has been added to the propofol to yield a concentration of 5 mcg/mL. Predicted plasma propofol concentration (solid green line) and effect‐site concentration (dashed green line). Predicted plasma remifentanil concentration (solid blue line) and effect‐site concentration (dashed blue line). The Paedfusor and Eleveld kinetic sets were used for propofol and remifentanil, respectively. Predicted effect‐site remifentanil concentration overshoots when the infusion is started and undershoots when the target concentration is decreased at 10 minutes

**Figure 3:**
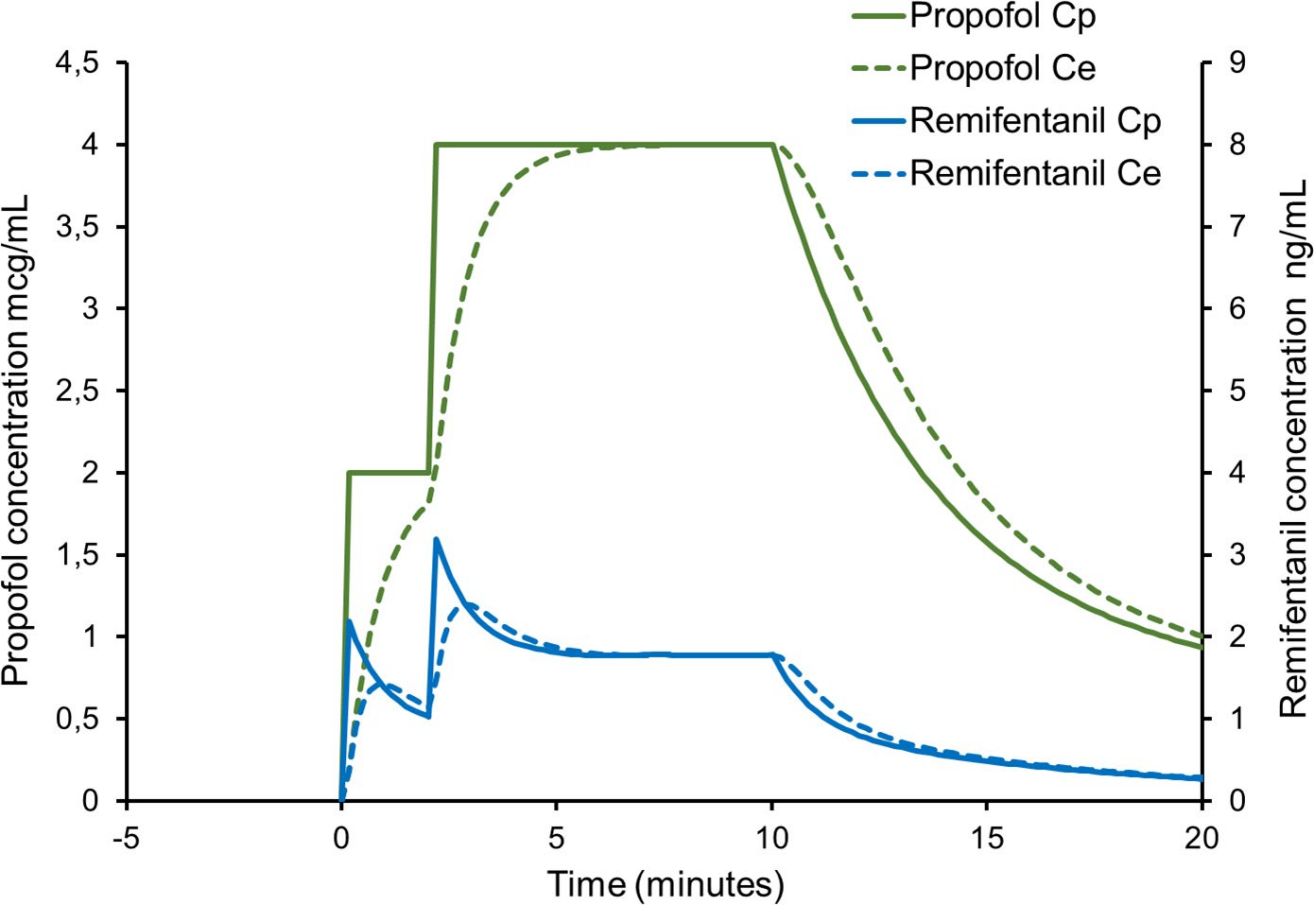
Simulated 10 minute TCI of propofol (target plasma concentration 2 mcg/mL for 2 minutes, thereafter 4 mcg/mL) for a 10‐year‐old male, weight 32 kg and height 140 cm using Stanpump software. Remifentanil has been added to the propofol to yield a concentration of 5 mcg/mL. Predicted plasma propofol concentration (solid green line) and effect‐site concentration (dashed green line). Predicted plasma remifentanil concentration (solid blue line) and effect‐site concentration (dashed blue line). The Paedfusor and Eleveld pharmacokinetic sets were used for propofol and remifentanil, respectively. Staging the approach to the initial target plasma concentration of propofol attenuates the relative overdosage of remifentanil

Figures 1, 2, 3 represent simulated infusions of this mix. To simplify the calculations, we have assumed a propofol concentration of 10 mg/mL (ie, 1%) and a remifentanil concentration of 5 mcg/mL in the mix. For each figure, we first used Stanpump software^1^ to calculate the infusion rates required for the simulated plasma target propofol concentration profile, with the Paedfusor model parameters for a 10‐year‐old male, weight 32 kg and height 140 cm. For illustration purposes, we also estimated the effect‐site propofol concentration using a k_e0_ of 0.26/min.^25^ We then programmed Stanpump with the Eleveld PK/PD model parameters for remifentanil for this child,^26^ to estimate the resulting estimated remifentanil plasma and effect‐site concentrations arising. Figure 1 shows that when the infusion is started at a target concentration of 4 mcg/mL, there is a rapid but temporary rise in remifentanil plasma and effect‐site concentrations. The peak effect‐site concentration is of the order of 5 ng/mL but thereafter it will plateau at around 2 ng/mL_._ Whereas 5 ng/mL is an appropriate concentration during painful procedures (such as laryngoscopy), the subsequent plateau concentration is at a level at which most patients will not breathe spontaneously, but will still move in response to painful stimuli. At present, clinically available TCI pumps do not allow effect‐site controlled TCI with the Paedfusor and Kataria models. Should this mode become available in the future, then clinicians should remember that the initial bolus is bigger with effect‐site targeting than with plasma targeting. In this case, if a TCI pump is used to administer an effect‐site targeted infusion of a propofol‐remifentanil mixture, the peak remifentanil concentrations will be substantially higher than 5 ng/mL, particularly if the TCI pump is programmed with a slow (low value) k_e0_.^21^

If the propofol target concentration is initially 4 mcg/mL but is decreased to 2.9 mcg/mL after 10 minutes (Figure 2) causing the infusion of the mixture to stop temporarily, the faster kinetics of remifentanil dictate that the remifentanil concentrations will fall proportionately more than the propofol concentration. In fact, the simulation shows that the estimated effect‐site remifentanil concentrations will fall to around 1 ng/mL a level that is insufficient to inhibit movement responses to pain. In essence, the simulation shows much less stable remifentanil plasma and effect‐site concentrations than propofol concentrations, with declines to inadequate remifentanil concentrations.

Since the kinetics of remifentanil are linear, the achieved plasma and effect‐site concentrations can be extrapolated from the above simulations. Mixtures containing less remifentanil will likely result in inadequate plasma and effect‐site concentrations, and this will be exacerbated by the instability of remifentanil over time in mixtures containing low remifentanil concentrations. On the other hand, more concentrated mixtures will result in very high peak effect‐site remifentanil concentrations (≥10 ng/mL) at which adverse effects are expected (bradycardia and hypotension) and plateau and nadir concentrations at which apnea will be invariable.

Feedback from clinicians and simulation suggest that the impact of the transient remifentanil overdosage at the start of TCI may be attenuated by staging the approach to the initial TCI target rather than achieving it in a single step (Figure 3).

## 9 CONCLUSION

Although pediatric anesthetists who administer propofol‐remifentanil mixtures are probably in good company, they should remember that this technique has several dangers, theoretical or otherwise, as described above. Clinicians should be aware that if they practice this technique, and a patient under their care suffers a critical incident, it is likely that their practice will come under close scrutiny. While they could offer as a defense the fact that there is a body of reasonable and experienced clinicians who practice this technique, it is unlikely that they will find support from health and regulatory bodies (such as the MHRA and the General Medical Council in the UK).

## CONFLICT OF INTEREST

ARA: His research group/department received grants and funding from The Medicines Company (Parsippany, NJ, USA), Becton Dickinson (Eysins, Switzerland), Dräger (Lübeck, Germany), Paion (Aachen, Germany), and Rigel (San Francisco, CA, USA); and he has received honoraria from The Medicines Company (Parsippany, NJ, USA), Janssen Pharmaceutica NV (Beerse, Belgium), Becton Dickinson (Eysins, Switzerland), Paion (Aachen, Germany), Rigel (San Francisco, CA, USA), Philips (Eindhoven, Netherlands), and Ever Pharma (Unterach, Austria). AER‐J: None. ARR: None. JRS: Consultant to Paion (Aachen, Germany) and Altus (Toronto, Canada).

## Footnotes

^1^ Freely available from the author at http://opentci.org/code/stanpump (accessed September 22, 2020).
